# Protective and proliferative effect of *Aesculus indica* extract on stressed human adipose stem cells via downregulation of NF-κB pathway

**DOI:** 10.1371/journal.pone.0258762

**Published:** 2021-10-22

**Authors:** Hamzah Khawaja, Numan Fazal, Faiza Yaqub, Muhammad Rauf Ahmad, Muzaffar Hanif, Muhammad Amin Yousaf, Noreen Latief

**Affiliations:** 1 National Centre of Excellence in Molecular Biology, University of the Punjab, Lahore, Pakistan; 2 Institute of Laboratory Medicine, Clinical Chemistry, and Molecular Diagnostics, Leipzig University, Leipzig, Germany; 3 Department of Molecular Biology, Shaheed Zulfiqar Ali Bhutto Medical University, Islamabad, Pakistan; 4 Department of Dermatology, Jinnah Burn & Reconstructive Surgery Centre, Lahore, Pakistan; Government College University Faisalabad, PAKISTAN

## Abstract

Inflammatory microenvironment after transplantation affects the proliferation and causes senescence of adipose-derived mesenchymal stem cells (hADMSCs) thus compromising their clinical efficacy. Priming stem cells with herbal extracts is considered very promising to improve their viability in the inflammatory milieu. *Aesculus indica* (*A*. *indica*) is used to treat many inflammatory diseases in Asia for decades. Herein, we explored the protective role of *A*. *indica* extract on human adipose-derived Mesenchymal Stem Cells (hADMSCs) against Monosodium Iodoacetate (MIA) induced stress *in vitro*. *A*. *indica* ameliorated the injury as depicted by significantly enhanced proliferation, viability, improved cell migration and superoxide dismutase activity. Furthermore, reduced lactate dehydrogenase activity, reactive oxygen species release, senescent and apoptotic cells were detected in *A*. *indica* primed hADMSCs. Downregulation of NF-κB pathway and associated inflammatory genes, NF-κB p65/RelA and p50/NF-κB 1, Interleukin 6 (IL-6), Interleukin 1 (IL-1β), Tumor necrosis factor alpha (TNF-α) and matrix metalloproteinase 13 (MMP-13) were observed in *A*. *indica* primed hADMSCs as compared to stressed hADMSCs. Complementary to gene expression, *A*. *indica* priming reduced the release of transcription factor p65, inhibitory-κB kinase (IKK) α and β, IL-1β and TNF-α proteins expression. Our data elucidates that *A*. *indica* extract preconditioning rescued hADMSCs against oxidative stress and improved their therapeutic potential by relieving inflammation through regulation of NF-κB pathway.

## 1. Introduction

Stem cell transplantation has become a popular therapy for diseases that are prone to cause tissue degeneration like, Crohn’s disease, chronic liver, kidney failure and rheumatoid arthritis [1]. However, it has been observed that during transplantation. Mesenchymal Stem Cells (MSCs) do not survive the harsh microenvironment of the inflammatory region, due to the hypoxia stress caused by reactive oxygen species produced by the inflammatory environment [2]. Exponential levels of ROS can hyper-trigger signaling cascades that encourage inflammation leading to cell death [3]. ROS stimulates the expression of inflammasomes through different downstream processes including the NF-κB pathway, which is considered to be the cellular ‘sensor’ for ROS-induced stress [4].

Nuclear Factor kappa-light-chain-enhancer of activated B cells (NF-κB) proteins are considered as the important transcription factors that regulate the innate and adaptive immune response [5]. The NF-κB is mainly regulated through five key members including c-Rel, p50/p105 (NF-κB1), Rel A (p65), p100/52 (NF-κB2) and Rel B [6]. Various upstream stimuli activate the NF-κB signals, however, in unstimulated conditions, two inhibitor kinases; IKK1 (IKKα), IKK2 (IKKβ) along with a regulatory subunit NEMO (IKKG), forms complexes with the NF-κB thereby keeping NF-κB inactive in the cytoplasm. After stimulated through activation signal, the phosphorylation of IkB occurs at two amino (N)-terminal regulatory serine residues leading to IkB ubiquitylation and subsequent degradation [7, 8]. This process initiates the release and translocation of NF-κB heterodimer from the cytoplasm to the nucleus activating the expression of associated genes [6].

Recently, inflammatory control of adipose mesenchymal stem cells has emerged as a novel mode of inflammatory regulation through ADMSCs. ADMSCs can sense and downregulate pro-inflammatory cytokines and modulate immune insults [9, 10]. ADMSCs possess active TLR2, TLR3, and TLR4, because activation with specific ligands resulted in the induction of NF-κB dependent genes including superoxide dismutase and the release of interleukin IL6 and IL-8 [11]. Furthermore, a range of chemokines and proinflammatory cytokines that include TNF-α, IL1β, IL6, IL17 and IFN-γ [12] has been shown to influence ADMSCs. Therefore, NF-κB can be regarded as a regulator of the inflammatory control of ADMSCs. NF-κB is considered as the major down regulator of signals transduced by both pro-inflammatory cytokines and TLRs [5, 6]. Besides, increased uncontrolled NF-κB activity upregulates the expression of the pro-inflammatory cytokines, including IL1, TNF, IL6, and IFNγ [13–16]. Stimulation of NF-κB signaling inhibits proliferation and adipogenic differentiation of hADMSCs [17]. Although these studies reveal the significance of NF-κB pathways in ADMSCs proliferation, the downstream consequences of NF-κB signals, and the precise mechanisms through which NF-κB controls hADMSCs remain largely unknown.

Different strategies are being applied to increase the survival ability of ADMSCs against high ROS levels in the degenerative environment, one such novel modus operandi considered is to precondition cells with different substrates having an affinity with mesenchymal stem cells [2]. Therefore, stem cells have been preconditioned with various bioactive molecules (Curcumin, VEGF, etc.) and transforming procedures like electrical stimulation and hypoxic pre-treatment to increase their homing potential [18–20]. One such preconditioning approach is to use plant extracts that have antioxidant properties [21].

Different plant extracts can counter oxidative stress and inflammation in a tissue environment [22–24]. One such herb, researched to have these key parameters is *Aesculus indica* (*A*. *indica*), as it inherits both antioxidant and anti-inflammatory properties [25, 26]. Potent anti-oxidant bioactive compounds like Mandelic acid and quercetin have been isolated from *A*. *indica*, whereas its potential to counter inflammatory conditions comes from its core molecule compound called aescin, which is the mixture of triterpenoid saponin which has a superb antiviral, antiangiogenic (vascular protection), antifungal, anti-obesity, antioxidant, anti-inflammatory, antigenotoxic and antitumor properties [27, 28]. Additionally, *A*. *indica* extract has been characterized and a wide range of phytochemicals including qurcetin (a standard flavonoid) has been reported previously [28, 29]. *A*. *indica* shows significant potential in dealing with oxidative and inflammatory stress environment, but its effect on aiding mesenchymal stem cells to survive harsh inflammatory environments conditions both *in vitro* and *in vivo* are still unknown.

## 2. Materials and methods

### 2.1 Collection and identification of plant material

Dried fruits of *A*. *indica* (Bankhor) used in this study were collected from Swat Pakistan. The specimen (Voucher # LAH35852) authenticity was confirmed by the herbarium of University of the Punjab, Lahore, Pakistan. Fruits were dried in the shade for one week at room temperature and then crushed into fine powder. The powder was weighed, suspended in 3X methanol and left for 2 days in a shaker (New Brunswick^™^ Innova^®^ 44/44R). The mixture was then filtered through Whatmann paper. The remaining residual was resuspended in 2× methanol and kept in shaker for 2 days. Afterward, the solution was filtered through Whatmann filter paper. The residue leftover was eventually suspended in 1× methanol and kept on shaker for 1 day and lastly filtered. The filtrate was pooled, evaporated to dryness and incubated at 37°C for 3 days to obtain the dried extract. The dry powdered extract was stored at 4°C for further utilization in experimental studies and phytochemical screening. Furthermore, the *A*. *indica* extract was sticky in nature, dark green in colour and had a chemical odour.

### 2.2 Phytochemical analysis and antioxidant determination

Folin–Ciocalteu reagent and Aluminium chloride colorimetric method was used to estimate the total phenolic content (TPC) and total flavonoid content (TFC) in the *A*. *indica* extract with slight modification [34]. The Gallic acid calibration curve was used as a standard to calculate the TPC, while quercetin calibration curve was used to calculate TFC, expressed as μg per equivalent mg of dry weight. Moreover, DPPH (2,2-diphenyl-1-picryl-hydrazyl-hydrate) radical scavenging technique was used to determine the free radical scavenging activity of *A*. *indica* extract [35].

### 2.3 Isolation and culturing of human Adipose-Derived Stem Cells (hADMSCs)

The study was approved and conducted in stem cells lab at the National Centre of Excellence in Molecular Biology (CEMB), Lahore, Pakistan following approval of Ethical Review Board (ERB) committee of CEMB. The adipose aspirate was collected from patients that were selected based on selection criteria, pre-requisitioning non-diabetic negative viral status young patients, undergoing normal cosmetic surgery. All biopsies (n = 10) were obtained from Jinnah burn and Reconstructive Surgery Centre (JBRSC), with written consent from the patients before surgery. hADMSCs were isolated according to a previously described protocol with slight modifications [36]. The collected sample was washed thrice with 1X PBS and treated with Collagenase 1A and incubated at 37°C for 45 minutes. Collagen was inactivated by adding 10% FBS containing media, filtered through a 100μm mesh and centrifuged for 10 minutes at 1200xg to obtain SVF in the form of a pallet. SVF was resuspended in 1ml media and shifted to a 75cm2 flask. We have previously reported the characterization of hADMSCs through Fluorescence-Activated Cell Sorting (FACS) [30].

### 2.4 Effect of *A*. *indica* on hADMSCs proliferation and viability

Pretreated hADMSCs with different concentrations of *A*. *indica* were assessed for their proliferation and viability by MTT and Trypan blue assay. For MTT, 8000cells/well hADMSCs were seeded in a 96 well plate and treated with different *A*. *indica* extract concentrations (1μg/ml, 5μg/ml, 10μg/ml, 20μg/ml, 40μg/ml, 60μg/ml) for 24 hours. Cell metabolic activity was measured after 24 hours at 570nm through a microplate reader. For Trypan blue exclusion assay, *A*. *indica* treated hADMSCs were resuspended in 1ml media after being trypsinized and mixed with 0.4% trypan blue in equal ratio to count live and dead cells. 10μg/ml and 20μg/ml *A*. *indica* doses were selected from analysis for further preconditioning experiments against MIA stress.

MIA stress of 10μM was used for inducing stress according to the protocol previously described [30]. Furthermore, hADMSCs were evaluated for their viability and metabolic activity to evaluate the effects of MIA-induced oxidative stress. Similarly, hADMSCsat passage 3 were plated in a 96 well plate (8000cells/ well) and preconditioned with *A*. *indica* extracts (10μg/ml and 20μg/ml) for 24hours and afterward treated with 10 μM MIA for 24 hours to evaluate the MIA stress mitigating effects of *A*. *indica* extract on hADMSCs proliferation and viability by performing MTT and Trypan blue exclusion assay.

### 2.5 Effect of *A*. *indica* on cell senescence

Cell senescence assay was performed according to the manufacturer’s protocol (Abcam, Cambridge, MA, USA). Briefly, hADMSCs were plated in 12 well plate (30000/ well) and preconditioned with *A*. *indica* extract (10μg/ml and 20μg/ml) and treated with 10 μM MIA for 24 hours respectively. hADMSCs were afterward washed with 1 X PBS and fixed with 0.5ml fixative solution for 15 min. Cells were washed twice with1 X PBS and stained with a staining solution overnight. hADMSCs were analyzed under Olympus BX61 microscope (Olympus America Inc. USA) at 100μm scale and 100x magnification for further analysis using ImageJ software.

### 2.6 Cell migration analysis using wound healing assay

*A*. *indica* extract preconditioned hADMSCs were assessed for their migration potential using scratch wound assay. Briefly, 8 x 104 cells were seeded in a 6-well plate and left to form a monolayer. Monolayers were scratched with a sterile plastic 200luL micropipette tip and washed with 1X PBS. Images were taken after 24hr, 48hr and 72hr time intervals at 500μm scale with 40x magnification. ImageJ software (National Institutes of Health, USA) was used for wound size reduction analysis.

### 2.7 Biochemical assays

Biochemical analysis was performed on the media collected from different hADMSCs groups (Control, Stress and *A*. *indica* preconditioned). The isolated media was analyzed for LDH levels (Roche Diagnostics, Cat No. 04744926001), Reactive oxygen species (ROS) (Abcam, ab113851DCFDA) and superoxide dismutase (Abcam USA, Cat No. ab65354) to evaluate the effect of *A*. *indica* on injured hADMSCs.

### 2.8 Semi-quantitative real-time polymerase chain reaction (PCR)

Total RNA was isolated from control, stress and preconditioned groups using trizol method. cDNA was reverse transcribed from isolated RNA (1μg) through cDNA synthesis kit (Thermo Scientific, Cat No: K1622). Primer sequences are listed in Table 1. All experiments were run in triplicate and mRNA levels of β actin were determined for the normalization of the p50, TNF-α, MMP13, IL-1β and P65 mRNA expression values.

**Table 1:** List of primers used in the study.

S. NO	Gene	Primer sequence (5′-------3′)	GC Content (%)	Annealing Tm (°C)
1	Tnf-α	F: TGCTTGTTCCTCAGCCTCTT	50	60
		R: ATGAGGTACAGGCCCTCTGAT	52	
2	NF-κB (P65)	F: CCACGAGCTTGTAGGAAAGG	55	59
		R: GCACAGCATTCAGGTCGTAG	55	
3	NF-κB P105	F: CTGGAAGCACGAATGACAGA	50	59
		R: GTCCATCTCCTTGGTCTGCT	55	
4	IL-1β	F: GCATCCAGCTACGAATCTCC	55	59
		R: CGTGCACATAAGCCTCGTTA	50	
5	IL-6	F: CTGCGTCCGTAGTTTCCTTC	55	59
		R: GAGGTGAGTGGCTGTCTGTG	55	
6	MMP-13	F: GCTGCCTTCCTCTTCTTGAG	55	59
		R: TCGTCAAGTTTGCCAGTCAC	55	
7	β-Actin	F: CGCATGGGTCAGAAGGATTC	55	59
		R: TAGAAGGTGTGGTGCCAGATTT	45	

### 2.9 Enzyme-linked immunosorbent assay

1 x 10^4^ cells were seeded in 6 well plate for all the groups (control, stress and *A*. *indica* Preconditioned). The cells were lysed using RIPA buffer containing Protease Inhibitor (Roche cat# 04693116001). The cell lysates were assessed for inflammatory cytokines and transcription factors using ELISA. The ELISA for IL-1 (Abcam cat # ab46052), TNF-α (Abcam cat # ab181421), NF-kappa (p65) (Abcam cat # ab133112) and IKKα and IKKβ (Cytoglow cat # CB5358) was performed using a commercially available kit, according to the manufacturer’s instructions.

### 2.10 Statistical analysis

Statistical analysis of all results was represented as mean ± standard deviation. Significant differences between the groups were determined by using one way ANOVA with Bonferroni’s test. A p-value of less than 0.05 was considered significant. Graph-pad Prism software was used for creating graphs (GraphPad, version 5.00, USA). Moreover, all the experiments were run in triplicates (n = 3).

## 3. Results

### 3.1 Total phenolic and total flavonoid contents in *A*. *indica*

Phenolic and flavonoid compounds have significant anti-oxidant potential as they donate their electron and capture the reactive oxygen species. The total phenolic content was observed to be 108.926μg/mg of Gallic acid equivalent (GAE). Total flavonoid content of *A*. *indica* extract was evaluated to be 128μg/mg. This result shows that *A*. *indica* extract has potent phenolic and flavonoid content that can induce antioxidant activity by scavenging reactive oxygen species.

### 3.2 *A*. *indica* exhibited high free radical scavenging capacity

DPPH free radical scavenging of *A*. *indica* extract was exhibited as IC50 = 18.959μg/mg in comparison to Ascorbic acid (IC50 = 35.614μg/mg) in concentration-dependent manner. This reveals that *A*. *indica* extract is 53% more potent than ascorbic acid in scavenging radical species.

### 3.3 *A*. *indica* boosted metabolic activity and viability of hADMSCs

**Fig 1:**
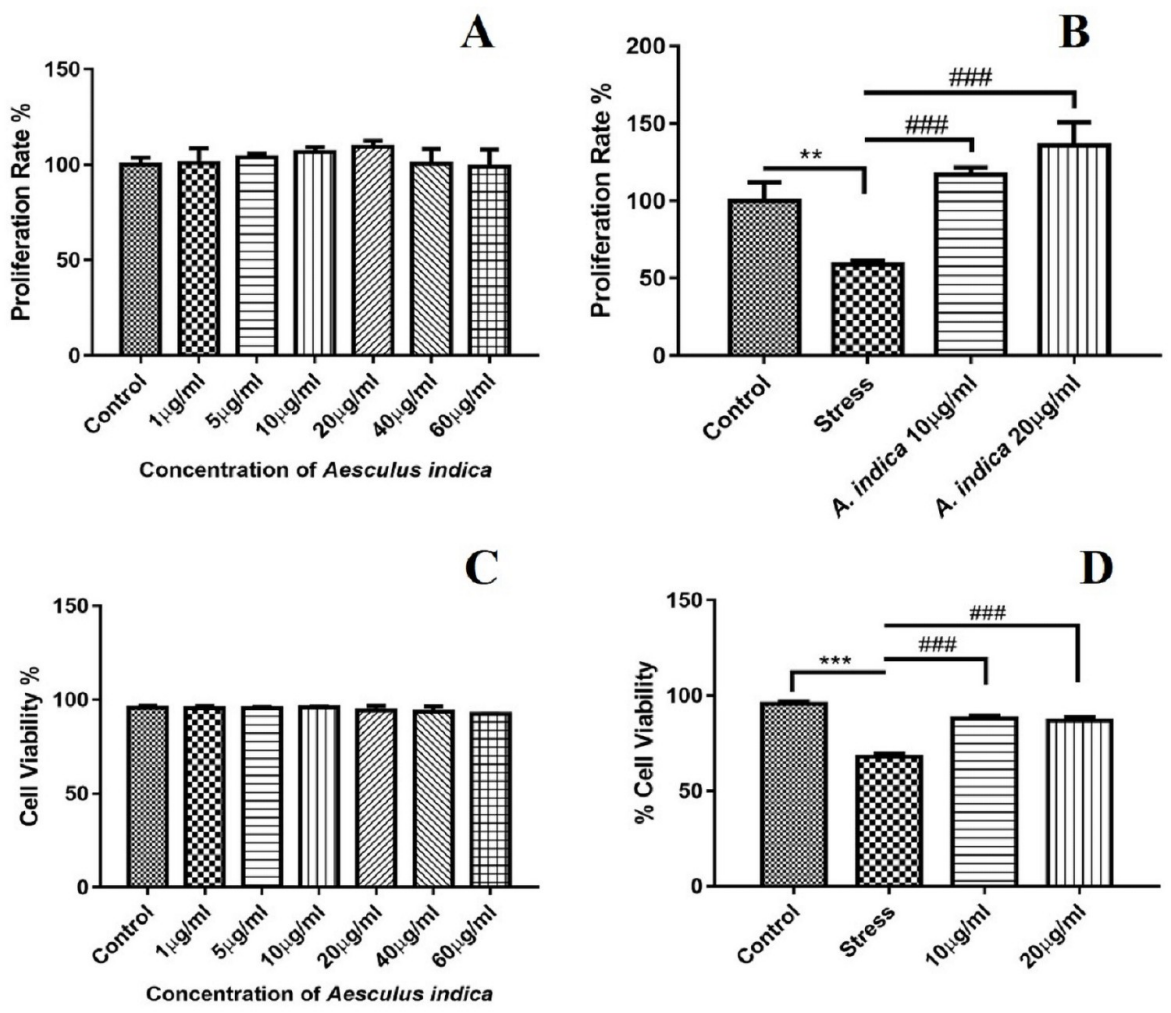
Bar graphs representing the metabolic activity and viability of hADMSCs in response to MIA stress and *A*. *indica* preconditioning. A. Metabolic activity of hADMSCs preconditioned with different doses of *A*. *indica* extract. B. Metabolic activity of MIA induced hADMSCs against different *A*. *indica* concentrations. C. Viability of hADMSCs preconditioned with *A*. *indica* extract concentrations. D. Percent viability of MIA induced hADMSC’s after *A*. *indica* preconditioning. Values are statistically significant at p*<0.05, p**< 0.01, p***< 0.001compared with the control group while p#<0.05, p##< 0.01, p###< 0.001 compared with stress group. The experiment was carried out in triplicates (n = 3).

Concentration of *A*. *indica* from 1μg to 60μg was assessed in comparison to control group, revealing that all the doses used were not toxic for the hADMSCs and that the maximum proliferation rate was observed in group treated with 20μg concentration of *A*. *indica* (Fig 1A and 1C).

### 3.4 *A*. *indica* preconditioning improved metabolic activity and viability in stressed hADMSCs

Cell proliferation and viability of *A*. *indica* preconditioned cells against MIA oxidative stress was assessed by observing metabolic activity and live cell count of hADMSCs. *A*. *indica* preconditioning with (10μg and 20μg) resulted in increased viability and proliferation of cells as compared to stress group (Fig 1B and 1D).

### 3.5 *A*. *indica* improved wound healing potential of hADMSCs

**Fig 2:**
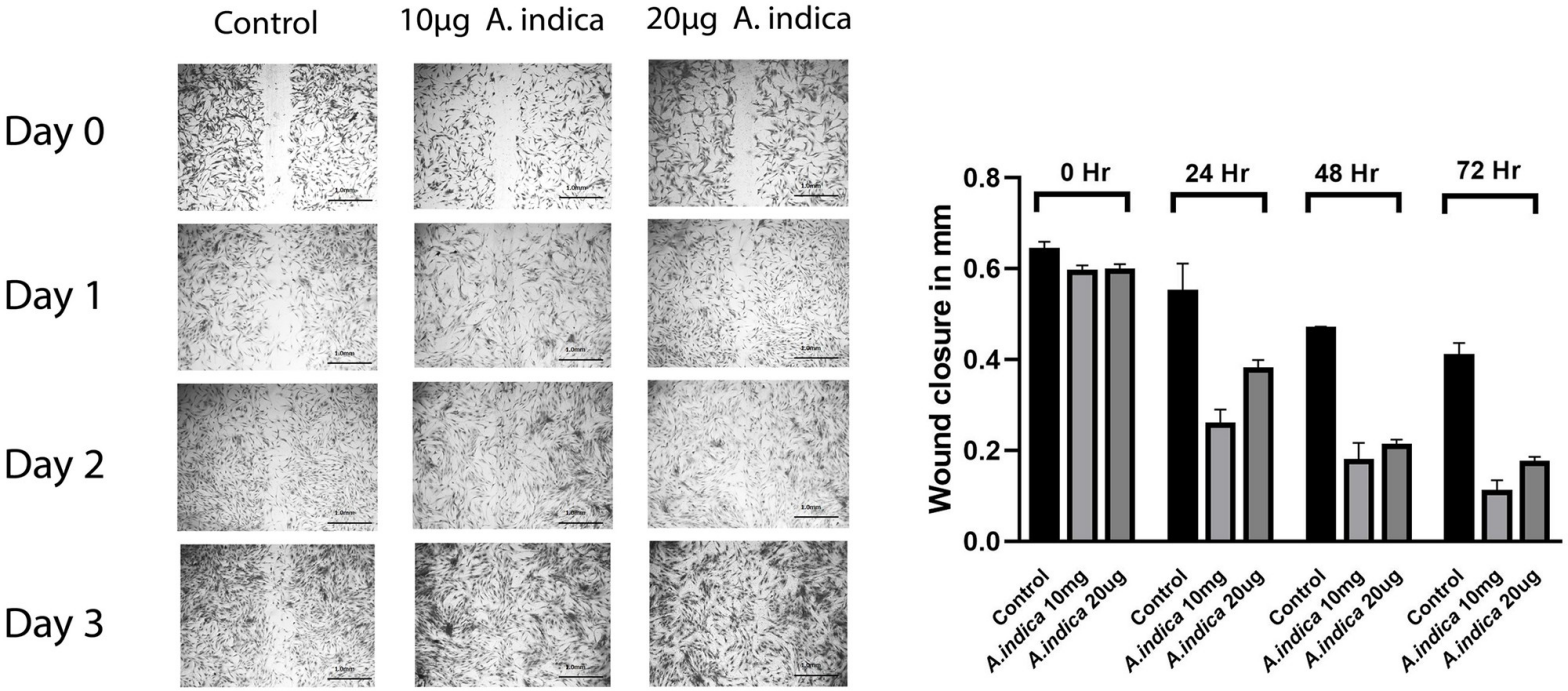
Wound healing assay of hADMSCs performed at 24hr time interval at 500μm scale and 40x magnification for three days where (A) represents the control and preconditioned hADMSCs (A.I represents *Aesculus indica*), while (B) represents the wound size in millimeter (mm) for three days at 24hrs time frame using ImageJ software.

High wound closure activity was observed in hADMSCs preconditioned with *A*. *indica* as compared to control group after 0hr, 24hr, 48hr and 72hrs. The wound was almost closed after 72hr in *A*. *indica* preconditioned group to control (Fig 2A and 2B).

### 3.6 *A*. *indica* reduced cell senescence in hADMSCs

**Fig 3:**
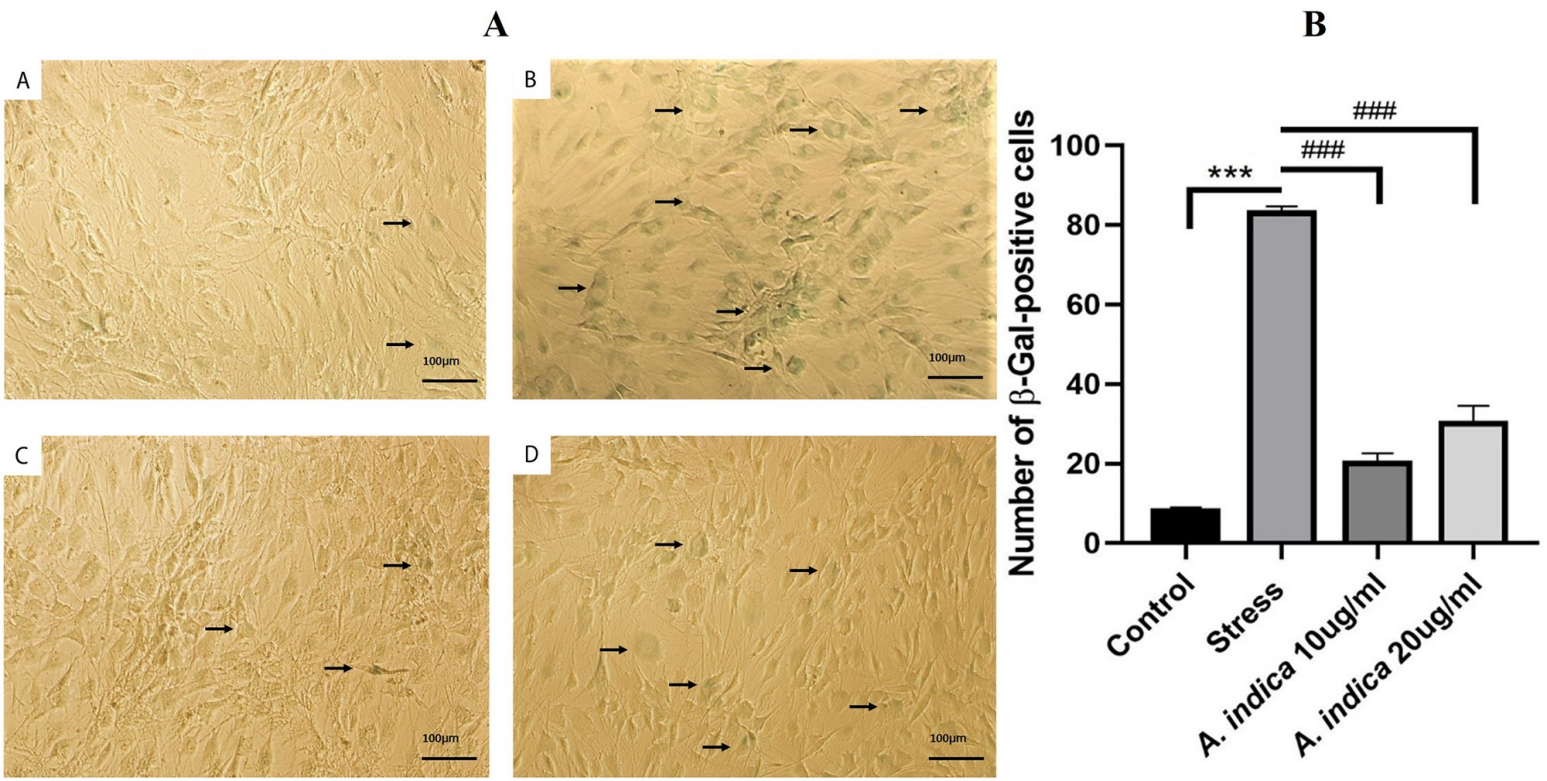
Cytoprotective effect of *A*. *indica* extract against MIA induced senescence in human adipose stem cells. A. (1) Control (2) 10μM MIA (3) 10μg and (4) 20μg *A*. *indica* extract preconditioned hADMSCs. Images were taken on at 100μm scale and 100x magnification. B. Bar graph represents number of senescence positive cells in each group. Analysis of number of senescence positive cells of each group was performed though ImageJ software while statistical analysis of the results was carried out through Prism software. Values are statistically significant at p*<0.05, p**< 0.01, p***< 0.001compared with the control group while p#<0.05, p##< 0.01, p###< 0.001 compared with stress group. The experiment was carried out in triplicates (n = 3).

Senescence is considered to be prerequisite sign of cell aging. The Senescence-associated beta-galactosidase (SA-β-Gal) can only be found in senescent cells and is not found in pre-senescent, quiescent or immortal cells. The Cells that were only given 10 μM MIA stress showed considerable amount of aged cells as compared to control and *A*. *indica* preconditioned (10μg and 20μg) groups (Fig 3A and 3B), (S1 Fig).

### 3.7 *A*. *indica* preconditioning decreased necrosis activity in stressed hADMSCs

**Fig 4:**
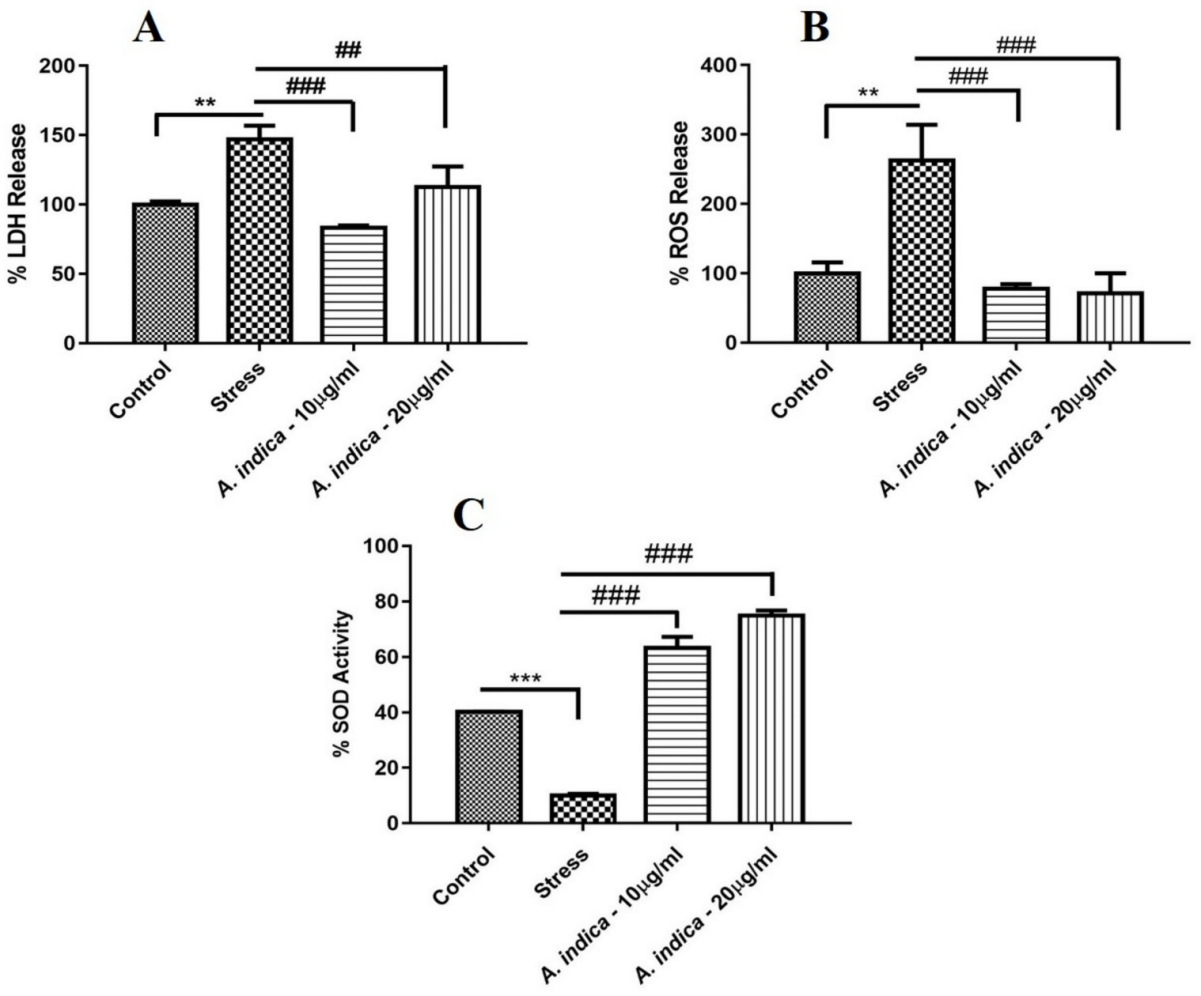
Effect of *A*. *indica* extract on LDH, ROS and SOD activity. (A) Activity of LDH in MIA induced hADMSCs preconditioned with A. indica extract concentrations. (B) Generation of ROS in hADMSCs preconditioned with *A*. *indica* extract and treated with MIA. (C) Percentage of SOD activity in MIA induced hADMSCs preconditioned with *A*. *indica* extract. Values are statistically significant at p*<0.05, p**< 0.01, p***< 0.001compared with the control group while p#<0.05, p##< 0.01, p###< 0.001 compared with stress group. The experiment was carried out in triplicates (n = 3).

Lactate dehydrogenase enzyme (LDH) is released as a result of cell membrane damage and cell death. Cell necrosis also leads to a high amount of LDH release. High LDH activity was observed in the stress group (10μM MIA), as compared to control and preconditioned hADMSCs groups after 24 hours of exposure. *A*. *indica* preconditioned hADMSCs showed significantly decreased levels of LDH even after exposure to 10μM MIA (Fig 4A) (S1 File).

### 3.8 *A*. *indica* alleviated ROS activity in injured hADMSCs

ROS molecules are produced as a consequence of different metabolic activities consuming oxygen molecules inside the cell. Cell death can occur if ROS concentrations break from the balance and oxidative stress develops, which can have deleterious effects on different cellular lipids, proteins and oxygen species. ROS activity was observed in all the groups. A high amount of ROS activity was observed in stressed hADMSCs (10μM MIA). Whereas ROS activity determined from the control group and the *A*. *indica* extract preconditioned groups was significantly low as compred to stress (Fig 4B).

### 3.9 *A*. *indica* enhanced Superoxide Dismutase (SOD) activity in hADMSCs

Superoxide Dismutase (SOD) is an oxidative enzyme located in the cytoplasm of the cell functionally triggered to reduce superoxide ions into less harmful products. SOD assay was performed on the conditioned media of experimental groups and it has been observed that the *A*. *indica* preconditioned cells exhibited more SOD activity as compared to the stress group (Fig 4C).

### 3.10 *A*. *indica* extract downregulated inflammatory and NF-κB associated markers

**Fig 5:**
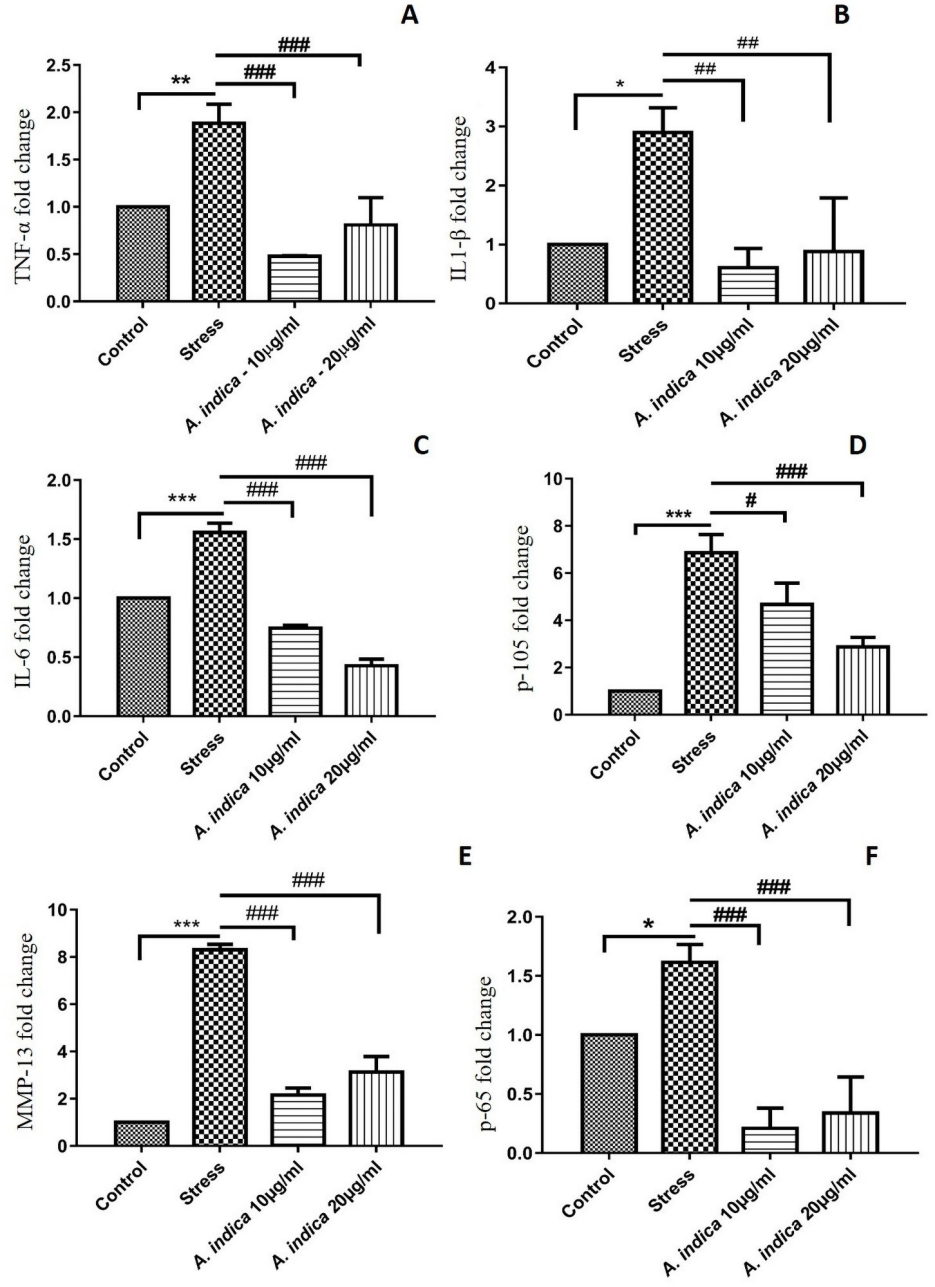
*A*. *indica* extract downregulated inflammatory and NF-κB associated markers. (A) TNF α (MIA-10μM: 1.884510 ± 0.2028108, *A*. *indica* 10μg/ml 0.4779985 ± 0.007028175, *A*. *indica* 20μg/ml 0.8084317 ± 0.2906602), (B) IL-1β (MIA-10μM: 2.899046 ± 0.4191191, *A*. *indica* 10μg/ml: 0.6099128 ± 0.3266602, *A*. *indica* 20μg/ml: 0.8838717 ± 0.9084732), (C) IL-6 (MIA-10μM: 1.556122 ± 0.07970555, *A*. *indica* 10μg/ml: 0.7459561 ± 0.0240984, *A*. *indica* 20μg/ml: 0.4277192 ± 0.05663558), (D) NF-κB p105(MIA-10μM: 6.866518 ± 0.7724251, *A*. *indica* 10μg/ml 4.671283 ± 0.9099906, *A*. *indica* 20μg/ml 2.872436± 0.4069114), (E) MMP-13(MIA-10μM: 8.303460 ± 0.2344566, *A*. *indica* 10μg/ml: 2.157991 ± 0.2970057, *A*. *indica* 20μg/ml: 3.130929 ± 0.6579115) and (F) NF-κB p65 (MIA-10μM: 1.614739 ± 0.1505901, *A*. *indica* 10μg/ml 0.2085242 ± 0.1716187, *A*. *indica* 20μg/ml: 0.3398924± 0.3039037) mRNA expression levels were determined by qPCR. Values are statistically significant at p*<0.05, p**< 0.01, p***< 0.001compared with the control group while p#<0.05, p##< 0.01, p###< 0.001 compared with stress group. The experiment was carried out in triplicates (n = 3).

Oxidative stress increases the release of inflammatory cytokines like Tumor necrosis factor alpha (TNF-α), Interleukin 6 (IL-6), Interleukin 1 (IL-1β), and matrix metalloproteinase 13 (MMP-13). Exposure to such inflammatory cytokines for a long period has shown to cause loss of self-renewal and quiescence. The levels of TNF-α, IL-6, IL-1β, and MMP-13 mRNA expression increased 1.88, 2.89, 1.55, 1.21 and 8.30 folds in the stress group respectively as compared to the control group and *A*. *indica* treated groups (Fig 5A, 5C, 5B and 5E). Similarly, the quantitative expression of Nuclear factor kappa-light-chain-enhancer of activated B cells (NF-κB) was measured by the mRNA level of NF-κB p65 and p50 (NF-κB 1) subunits in all the groups. The mRNA level of p65 and p50 markers increased 1.61 and 6.86 folds in the MIA induced stress group as compared to the control and *A*. *indica* treated groups (Fig 5D and 5F).

### 3.11 *A*. *indica* affected *the* pro-inflammatory cytokines production and NF-κB transcription factors protein expression

**Fig 6:**
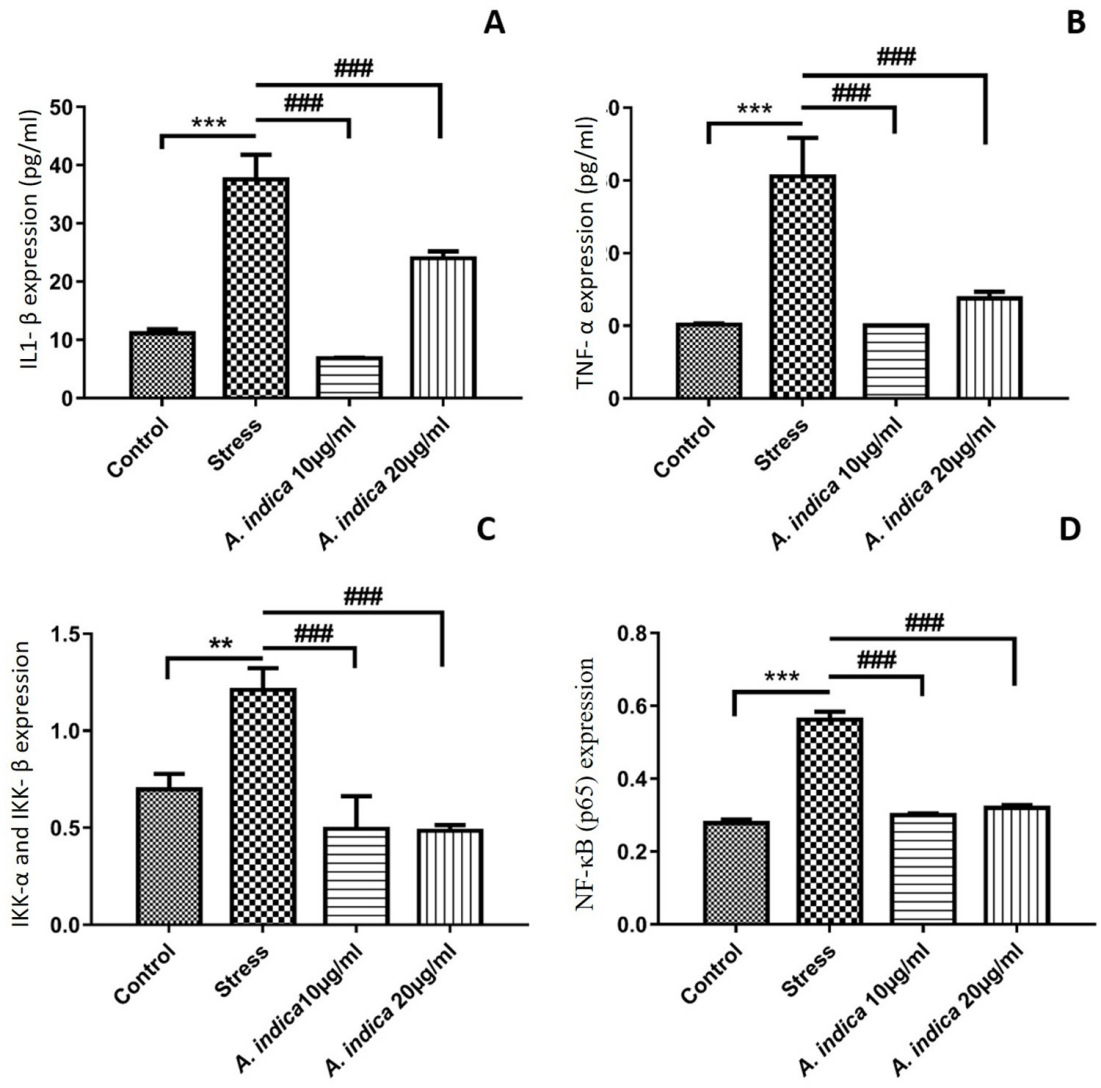
*A*. *indica* affected the proinflammatory cytokines production and NF-κB transcription factors protein expression. Protein expression levels of (A) IL-1 β (B) TNF α (C) IKK α and β and (D) NF-κB p65 were determined by ELISA. Values are statistically significant at p*<0.05, p**< 0.01, p***< 0.001compared with the control group while p#<0.05, p##< 0.01, p###< 0.001 compared with stress group. The experiment was carried out in triplicates (n = 3).

NF-κB has been frequently reported to cause the production and release of inflammatory cytokines like IL-1 and TNF-α in stem cells. In order to study the downregulation of inflammatory cytokines secretion, we evaluated NF-κB regulating factors and associated cytokines activity through. The p65 and inhibitory-κB kinase (IKK) α and β were found to be significantly elevated in MIA (10μM) stressed hADMSCs as compared to control and *A*. *indica* extract preconditioned groups (10μg and 20μg). Similarly, the production and concentration of IL-1β and TNF-α in nuclear extracts was calculated to be significantly high in 10μM MIA exposed hADMSCs. Whereas, hADMSCs in control group and A. *indica* preconditioned groups (10μg and 20μg) showed substantially less IL-1β and TNF-α protein expression (Fig 6).

## 4. Discussion

The use of mesenchymal stem cells has become a popular strategy for treating different conditions in quest of producing such therapy that provides the impaired tissues a regenerative potential without being affected by the inflammatory environment of the damaged tissue [1]. Various procedures to alter the stressful conditions that stem cells have to encounter after transplantation into inflammatory tissue are being researched by scientists worldwide including preconditioning cells with different substrates carrying affinity with mesenchymal stem cells [2]. One of the approaches is to use plant extracts that have anti-inflammatory and antioxidant properties [21]. *A*. *indica* is uniquely significant as it has been used to treat several painful conditions in the Asian subcontinent for centuries and has potent anti-inflammatory and antioxidant properties [31].

The present study elucidates the protective and proliferative effect of *A*. *indica* fruits extract in human adipose stem cells (hADMSCs) and its involvement in the downregulation of NF-κB pathway thereby relieving inflammation. *A*. *indica* treatment increased hADMSCs viability and metabolic activity in a dose-dependent manner as compared to *A*. *indica* untreated hADMSCs (Fig 1A and 1D). The efficacious effect of *A*. *indica* on cell viability was in correspondence with the previous research as *A*. *indica* is reputed to have several pragmatic benefits towards various biological cascades because of its antioxidant, antibacterial and anti-rheumatic properties [25, 26, 32]. Furthermore, MIA stress causes an increase in reactive oxygen species (ROS) due to oxidative stress which ultimately leads to cell death [33]. It was observed that MIA causes oxidative stress in human-derived chondrocytes and significantly affects that cellular integrity [34]. We used a similar MIA concentration (0.01 mg/mL) previously used to optimize oxidative stress on human chondrocytes and hADMSCs [30, 34].

Furthermore, MTT assay and trypan blue exclusion assay was performed to further evaluate the effect of *A*. *indica* preconditioning on stressed hADMSCs. The study revealed that the metabolic activity and cellular viability of hADMSCs were significantly preserved even after MIA treatment (Fig 1B and 1D). Related work has been performed previously on Tinospora cordifolia fruits, Mori rammulus, Mori fructus and Withania somnifera root extracts, as these extracts increased the viability and proliferation of the MSCs [35, 36]. Assessment of wound healing potential after 3 days with a 24 hours time interval revealed an increased proliferation rate in *A*. *indica* treated hADMSCs as compared to untreated cells (Fig 2). A similar study reported that Mori Fructus and Mori Ramulus have anti-oxidant properties which cause increased growth of mesenchymal stem cells to aid in the healing process respectively [36]. Moreover, cell scensense assay revealed more beta-galactosidase (SA-β-gal) activity in stressed hADMSCs as compared to *A*. *indica* preconditioned group (Fig 3A and 3B), our study affirms the protective role of *A*. *indica* as such decreased beta-galactosidase activity in human cells treated with various plant extracts has been reported previously [37].

Reports reveal that a high LDH level in the blood indicates acute or chronic cell damage [38]. We observed that the hADMSCs released more LDH content in the medium under hypoxic conditions than the cells pretreated with *A*. *indica* before the induction of hypoxia (Fig 4A). Similarly, a cell undergoes oxidative stress when reactive oxygen species (ROS) increases from normal level, which consequently damages cellular lipids, proteins and DNA content [39, 40]. As ROS release is directly proportional to an increase in oxidative stress, our findings suggested that *A*. *indica* extract was able to protect the hADMSCs against hypoxia-mediated cytotoxicity by scavenging reactive oxygen species (Fig 4B), as it’s already been reported that *A*. *indica* seeds have potent antioxidant potential to scavenge reactive oxygen species [41]. Oppositely, SOD enzyme present in the cells exhibit antioxidant property by catalyzing superoxide anion into hydrogen peroxide (H_2_O_2_) and molecular oxygen (O_2_) [42, 43]. SOD activity to combat oxidative stress is also significantly affected by the MIA concentration [44]. Elevated SOD activity in the *A*. *indica* pretreated cells further reinforced the antioxidant potential of *A*. *indica* against hypoxia in hADMSCs by hindering hypoxia-mediated apoptosis in the cells (Fig 4C). Hence, *A*. *indica* extract can also be implied to induce antioxidant potential in hADMSCs against hypoxia mediated inflammation regulated through NF-κB pathway.

Downregulation of NF-κB pathway can lead to decrease in oxidative stress where p65 takes part in NF-κB activation of several inflammatory markers. MIA oxidative stress increase NF-κB subunit p65 expression, stimulating NF-κB pathway mediated inflammation [45]. An increase in oxidative stress leads to increased activation of NF-κB cascade due to over activation of NF-κ B initiating markers including p105 [42, 43]. The downregulation in the expression of NF-κB subunit p65 in preconditioned hADMSCs showed that *A*. *indica* extract made the cells express less inflammatory marker and resist hypoxia-induced damage, as the decrease in p65 and p105 subunit expression at gene (Fig 5D and 5F) and protein levels (Fig 6D) is associated with less activity of NF-κB cascade, it leads to less production of inflammatory factors that would hinder the hADMSCs capability to resist oxidative stress [46, 47]. Furthermore, the IKKα and IKKβ serves as a key regulator of NF-κB (non-canonical) pathway as they phosphorylates the IκB protein which binds the p65 and p50 heterodimer thus releasing the heterodimer resulting in regulation of NFκB pathway [48, 49]. We observed decreased expression of IKKα and IKKβ proteins in *A*. *indica* preconditioned hADMSCs that suggests the inhibitory potential of *A*. *indica* as the downregulation of IKKα and IKKβ proteins has been previously reported in *Populus deltoides* treated RAW 264.7 cell lines [50].

As NF-κB positively regulates genes encoding cytokines (IL-1β, TNF-α, IL-6, etc) that are directly involved in inflammation [51]. Hence, the expression of these markers was studied at mRNA and protein levels. NF-κB is a key mediator that directly regulates TNF- α expression [52]. Different plant formulations like; Grape seed proanthocyanidin extract and GCSB-5 have evaluated for their potency to downregulate TNF-α expression [53, 54]. Therefore, the downregulation of TNF-α suggests that *A*. *indica* extract preconditioned hADMSCs resist the MIA mediated oxidative stress and hypoxia-mediated apoptosis (Figs 5A and 6B). Similarly, IL-1 is vital for the NF-κB pathway to interplay with inflammasome systems and IL-1 itself is regulated by the NF-κ B pathway. High levels of IL-1 in NF-κB activated inflammatory cascades, signifies its importance in NF-κB pathways. It has been observed that MIA is an exclusive activator of IL-1 gene expression [55, 56]. Our investigation revealed that *A*. *indica* pretreated cells significantly limit the expression of IL-1 as compared to the hypoxic cells (Figs 5B and 6A). The reduced expression of IL-1 in each *A*. *indica* pretreated experimental group strongly indicates the protective role of *A*. *indica* against hypoxic hADMSCs by allowing to perform their specific metabolic activities and hence enhancing hADMSCs regeneration capacity. In addition to IL-1, IL-6 is also actively involved in boosting inflammation process [57]. NF-κB facilitates the induction of IL-6 [58]. Herein, an increase in IL-6 was noted in stressed hADMSC’s as compared to *A*. *indica* pretreated experimental group (Fig 5C). Furthermore, a decrease in MMP-13 was also prominent in *A*. *indica* pretreated hADMSCs in comparison to stressed hADMSCs (Fig 5E). Active role of MMP-13 has been reported in inflammatory conditions, activation of TNF proinflammatory marker and IL-1β regulation [59–61].

## 5. Conclusion

The present study concludes that *A*. *indica* extract is a potent and potentially effective preconditioning tool in rescuing the hADMSCs against oxidative stress produced during inflammatory conditions and possesses valuable antioxidant and anti-inflammatory potential. The oxidative stress alleviating ability indicates that *A*. *indica* can be implied to treat ROS mediated inflammatory conditions. Moreover, the downregulation of NF-κB and associated markers (TNF-α, IL-1, IL-6) suggests the inhibitory potential of *A*. *indica* in relieving inflammation and hindering the progression of pathological conditions thereby increasing the chances of hADMSCs survival in the inflammatory microenvironment.

## Supporting information

S1 Fig Human adipose-derived mesenchymal stem cells senescence. 10.6084/m9.figshare.16352139.
(JPG)

S1 File Monosodium iodoacetate dose optimization in human adipose-derived mesenchymal stem cells. 10.6084/m9.figshare.16358268.
(PZF)
