# Supplementary figures and images for: Protective and proliferative effect of Aesculus indica extract on stressed human adipose stem cells via downregulation of NF-κB pathway
Source: PLoS One. 2021 Oct 22;16(10):e0258762. doi: 10.1371/journal.pone.0258762 (PMC8535185; doi:10.1371/journal.pone.0258762)

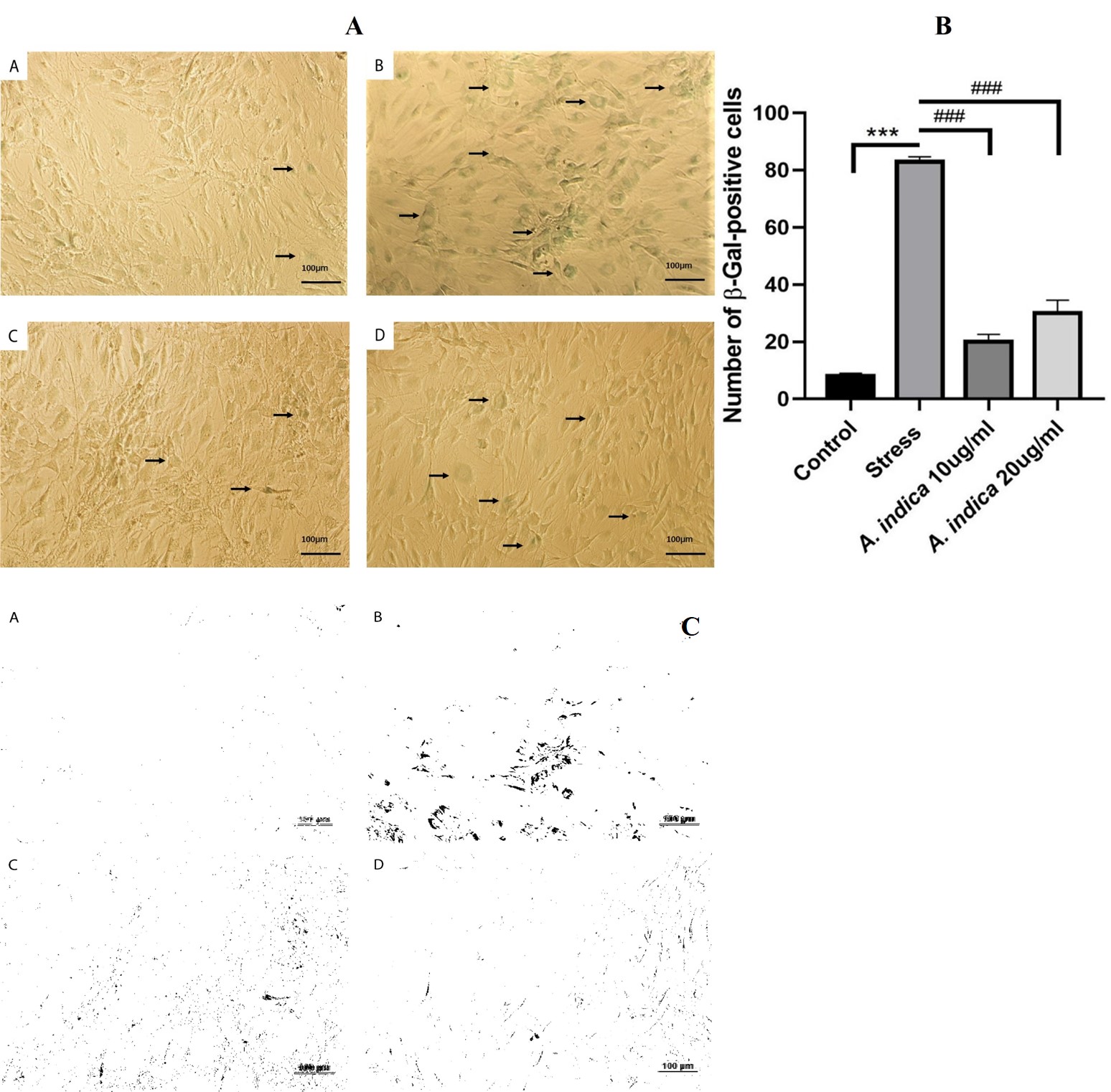

Supplement: S1 Fig — 10.6084/m9.figshare.16352139. (JPG) [file pone.0258762.s001.jpg]
